# Early‐life correlates of AD‐vulnerable relational memory in the structure and function of hippocampal subfields: Findings from the PRANK study

**DOI:** 10.1002/alz70856_103203

**Published:** 2025-12-24

**Authors:** Meghan K Ramirez, Connor J Phipps, Abi Heller, Jennifer N Sexton, Anna F Wilhelm, David E Warren

**Affiliations:** ^1^ University of Nebraska Medical Center, Omaha, NE, USA

## Abstract

**Background:**

Alzheimer's disease (AD) involves progressive neurodegeneration, especially in the hippocampus including early atrophy of the cornu ammonis 1 (CA1) subfield. While AD typically manifests in late life, vulnerability to AD may be associated with early‐life differences in hippocampal structure and function. Furthermore, other factors known to modulate brain development such as physical activity and socioeconomic status can affect AD risk. The periadolescent period, marked by heightened brain plasticity, may be particularly sensitive to these influences, shaping long‐term cognitive outcomes and late‐life AD vulnerability.

**Method:**

Cross‐sectional data were drawn from the Polygenic Risk of Alzheimer's disease in Nebraska Kids (PRANK) study of typically developing children (age 8‐13 years). Participants completed a relational memory task, studying pairs of objects while fMRI‐BOLD data were collected, then tested on memory for studied pairs. The task measured study‐time activation in brain regions linked to relational subsequent memory (RSM). The MRI protocol was adapted from the Human Connectome Project (HCP) Development/Aging study, including a T2‐weighted quasi‐coronal slab orthogonal to the long axis of the hippocampus (voxel size=.4 x 4 x2 mm; slices=30) for subfield segmentation. Data were preprocessed using the HCP minimal pipeline, and subfield volumes, including CA1, were measured with Automatic Segmentation of Hippocampal Subfields (ASHS) software. Task‐related RSM activity was interpreted using subfield masks. Participants also completed the KIDSCREEN‐52 questionnaire.

**Result:**

Successful RSM was associated with increased activity in right anterior CA1 (*p* < 0.001). Additionally, right CA1 volume was positively correlated with RSM (r=0.20, *p* = 0.02). We also examined the impact of self‐reported physical and financial well‐being on CA1 volume. Physical well‐being was negatively correlated with both left (r=‐0.46, *p* = 0.002) and right (*r* = ‐0.31, *p* = 0.046) CA1 volumes, while financial well‐being was negatively associated with both left (r=‐0.50, *p* = 0.001) and right (r=0.47, *p* = 0.002) CA1 volumes.

**Conclusion:**

These findings suggest that the CA1 is important for successful RSM performance. Furthermore, early‐life factors, such as physical and financial well‐being, may be associated with volume of the AD‐vulnerable CA1 subfield. Further investigation into these relationships could offer valuable insights into the role of periadolescent brain development in shaping late‐life AD risk.

